# Single Cell Analysis of Drug Susceptibility of *Mycobacterium abscessus* during Macrophage Infection

**DOI:** 10.3390/antibiotics9100711

**Published:** 2020-10-17

**Authors:** Joanna Brzostek, Amierah Fatin, Wen Hui Chua, Hui Yi Tan, Thomas Dick, Nicholas R. J. Gascoigne

**Affiliations:** 1 Department of Microbiology and Immunology, Yong Loo Lin School of Medcine, National University of Singapore, 5 Science Drive 2, Singapore 117545, Singapore; a0131634@u.nus.edu (A.F.); a0115583@u.nus.edu (W.H.C.); a0115342@u.nus.edu (H.Y.T.); thomas.dick@hmh-cdi.org (T.D.); 2 Center for Discovery and Innovation, Hackensack Meridian Health, Nutley, NJ 07110, USA; 3 Department of Medical Sciences, Hackensack Meridian School of Medicine at Seton Hall University, Nutley, NJ 07110, USA; 4 Immunology Programme, Life Sciences Institute, National University of Singapore, Singapore 117545, Singapore

**Keywords:** *Mycobacterium abscessus*, drug screen, single cell analysis

## Abstract

*Mycobacterium abscessus* is an emerging health risk to immunocompromised individuals and to people with pre-existing pulmonary conditions. As *M. abscessus* possesses multiple mechanisms of drug resistance, treatments of *M. abscessus* are of poor efficacy. Therefore, there is an urgent need for new therapeutic strategies targeting *M. abscessus*. We describe an experimental system for screening of compounds for their antimicrobial activity against intracellular *M. abscessus* using flow cytometry and imaging flow cytometry. The assay allows simultaneous analysis of multiple parameters, such as proportion of infected host cells, bacterial load per host cell from the infected population, and host cell viability. We verified the suitability of this method using two antibiotics with known activity against *M. abscessus*: clarithromycin and amikacin. Our analysis revealed a high degree of infection heterogeneity, which correlated with host cell size. A higher proportion of the larger host cells is infected with *M. abscessus* as compared to smaller host cells, and infected larger cells have higher intracellular bacterial burden than infected smaller cells. Clarithromycin treatment has a more pronounced effect on smaller host cells than on bigger host cells, suggesting that heterogeneity within the host cell population has an effect on antibiotic susceptibility of intracellular bacteria.

## 1. Introduction

*Mycobacterium abscessus* is a non-tuberculous mycobacteria (NTM) species, an opportunistic pathogen that mainly causes a tuberculosis-like lung disease in immunocompromised individuals and in people with pre-existing pulmonary conditions, such as cystic fibrosis [1]. The incidence of *M. abscessus* infections is rising worldwide [2], and *M. abscessus* is the most prevalent NTM species in patients in some parts of Asia [3,4,5,6]. *M. abscessus* infections have been thought to result from independent acquisitions of environmental bacteria. However, recent analyses of *M. abscessus* sequence diversity provide evidence for genetic clustering of patient isolates, suggesting human-to-human transmission [7,8].

*M. abscessus* infections can be mis-diagnosed as tuberculosis (TB, caused by *M. tuberculosis*) [9]; but *M. abscessus* is resistant to first-line anti-TB drugs [10]. *M. abscessus* employs multiple intrinsic mechanisms of drug resistance, including a cell envelope impermeable to hydrophilic antibiotics, expression of efflux pumps, as well as enzymes that degrade or modify antibiotics such as β-lactam-targeting β-lactamase or aminoglycoside phosphotransferases targeting aminoglycosides [10,11,12]. Consequently, treatment of *M. abscessus* infections is lengthy, often associated with side effects, and of very poor efficacy [13].

Given the increasing disease burden and lack of effective treatment options, there is an urgent need for new compounds targeting *M. abscessus* [14]. This requires improved drug screening strategies, as well as a better understanding of the biology of *M. abscessus* infection. Effects of anti-microbial agents on *M. abscessus* growth in liquid culture have been tested using standard microbiology techniques [15,16,17], and with fluorescent and luminescent reporters [18,19]. However, this experimental system does not recapitulate the physiology of *M. abscessus* survival and growth under conditions of limited nutrient availability. To overcome this limitation, metabolic activity of non-replicating *M. abscessus* in liquid culture under nutrient starvation was measured as a readout of bacterial viability under physiological conditions [20].

Importantly, drug susceptibility of extracellular *M. abscessus* does not always correlate with drug susceptibility in vivo. Drug screens using intracellular *M. abscessus* are therefore critical for development of better therapeutic strategies. *M. abscessus* infection can be established in immunocompromised mouse models [21], and the efficacy of several antimycobacterial drugs has been tested in this experimental system [22]. Alternative in vivo models for screening of compounds targeting *M. abscessus* have also been developed using zebrafish embryos [23] and fruit flies [24]. Here, we present an experimental system for rapid and simple drug screening during intracellular *M. abscessus* infection in macrophage cell lines, using flow cytometry and imaging flow cytometry.

Recent developments in single cell analysis techniques have revealed previously under-appreciated heterogeneity of infection at the single cell level. For example, influenza infection in cell lines results in very wide cell-to-cell variation in viral mRNA quantity, with viral mRNA constituting from less than 0.1% to half of the transcriptome of the infected cells [25]. Use of a dual fluorescence reporter system showed great variation in replication rates of intracellular *Salmonella* [26], and the presence of a subpopulation of non-dividing *M. tuberculosis* within infected macrophages [27]. Single cell RNA sequencing analyses have revealed a high degree of variation in gene expression, as well as in activation of innate immunity signaling pathways in host cells [28]. However, single cell heterogeneity during *M. abscessus* infections has not been previously investigated. In this study, use of flow cytometry-based methods for *M. abscessus* infection analysis at the single cell level allowed us to correlate morphological features of the host cells with bacterial burden.

## 2. Results

### 2.1. Fluorescent M. abscessus Reporter System in Macrophage Infections

In order to establish an experimental system to report *M. abscessus* intracellular infections, we cloned the gene encoding the mCherry fluorescent protein into the integrative mycobacterial expression vector pMV306hsp [29]. The *M. abscessus* Bamboo strain [30] was then transformed with the pMV306hsp-mCherry construct. The transformed strain will be referred to as *M. abscessus*-mCherry, and the parental Bamboo stain will be referred to as non-FL *M. abscessus. M. abscessus* Bamboo strain is a smooth colony morphotype derived from a clinical isolate. We have chosen the Bamboo strain as its whole genome has been sequenced [30], it has been used as a screening strain for *M. abscessus* drug discovery [16], and the strain was characterized in a range of “persister assays” [31].

**Figure 1:**
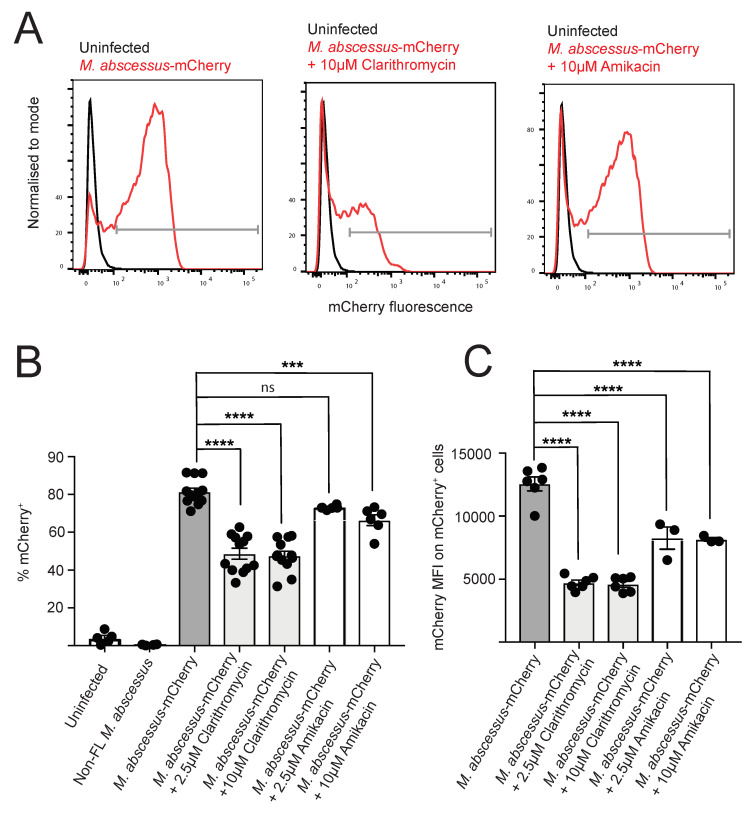
Single cell analysis of *M. abscessus*-mCherry infection and drug susceptibility using conventional flow cytometry. J774 macrophages were infected with *M. abscessus*-mCherry for 4 h, followed by 24 h culture with or without the indicated antibiotics. (**A**) Representative flow cytometry plots, (**B**) % of mCherry positive cells, and (**C**) mCherry MFI on mCherry^+^ cells. Data from 4 independent experiments (% mCherry^+^ untreated and clarithromycin-treated samples), 2 independent experiments (% mCherry^+^ amikacin-treated samples; mCherry MFI untreated and clarithromycin-treated samples, these are representative of 4 independent experiments) and 1 experiment (mCherry MFI amikacin-treated samples; this is representative of 2 independent experiments), with 3 technical repeats per experiment. One-way ANOVA with Tukey’s multiple comparisons test was used for statistical analysis. ns = *p* > 0.05, *** = *p* ≤ 0.001, **** = *p* ≤ 0.0001.

**Figure 2:**
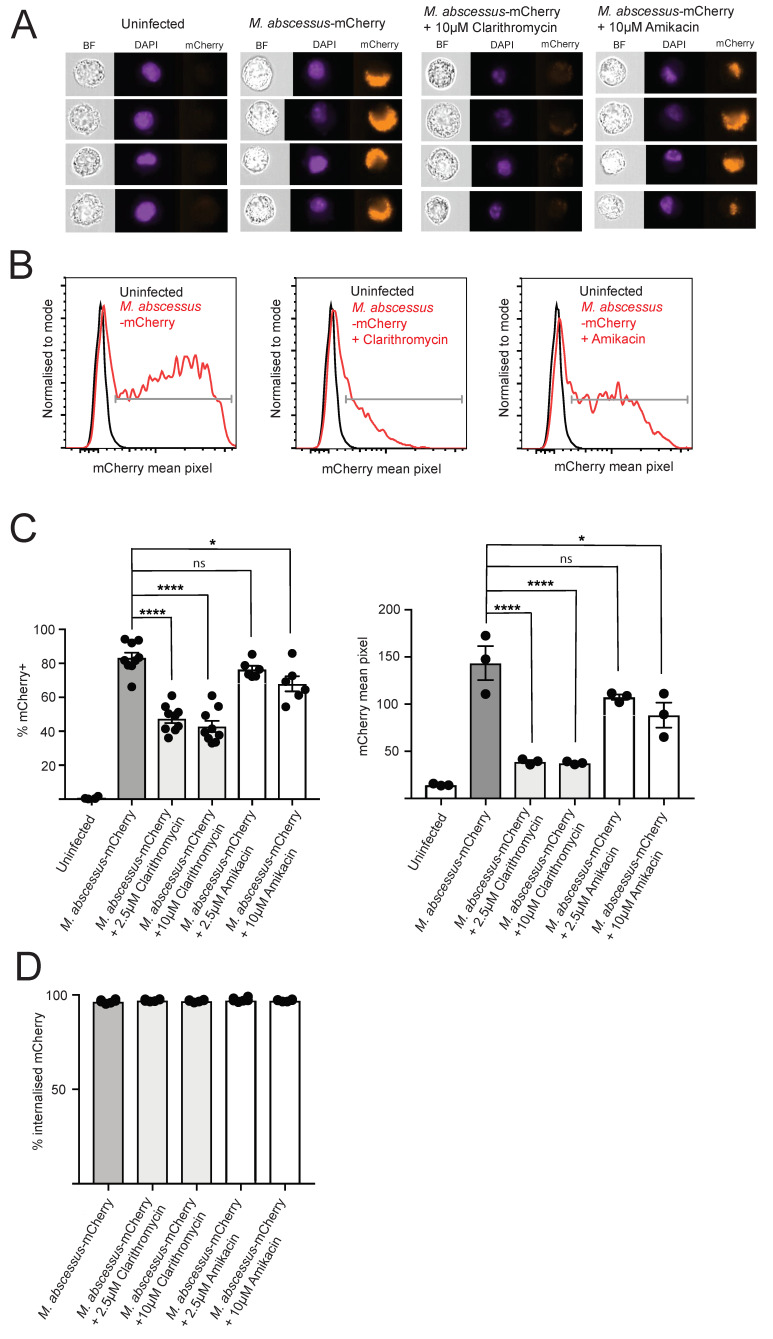
Single cell analysis of *M. abscessus* + mCherry infection and drug susceptibility using imaging flow cytometry. J774 macrophages were infected with *M. abscessus* + mCherry for 4 h, followed by 24 h culture with or without the indicated antibiotics for 24 h. (**A**) Representative images showing brightfield image, nuclear (DAPI) stain and mCherry image. (**B**) Representative plots of mCherry mean pixel fluorescence. (**C**) % of mCherry^+^ cells and mCherry mean pixel fluorescence on the mCherry^+^ population. (**D**) % of mCherry^+^ cells with internalized mCherry signal. Data from 3 independent experiments (% mCherry^+^ untreated and clarithromycin-treated samples), 2% mCherry^+^ amikacin-treated samples) and 1 experiment (mCherry mean pixel, this is representative of 3 experiments for clarithromycin-treated samples, and 2 experiments with amikacin-treated samples), with 3 technical repeats per experiment. One-way ANOVA with Tukey’s multiple comparisons test was used for statistical analysis. ns = *p* > 0.05, * = *p* ≤ 0.05, **** = *p* ≤ 0.0001.

Murine macrophage cell line J774 was infected with *M. abscessus*-mCherry or control non-FL *M. abscessus* for 4 h, followed by extensive washing to remove extracellular bacteria. The infected macrophages were then incubated for 24 h, followed by live/dead cell staining and analysis by conventional flow cytometry and imaging flow cytometry. At this time point of post-infection analysis, the macrophage viability was not affected by infection (Figure S1). As determined by flow cytometry, infection with *M. abscessus*-mCherry resulted in the appearance of an mCherry^+^ macrophage population (Figure 1A), with more than 80% of cells showing mCherry fluorescence values higher than those of uninfected macrophages or macrophages infected with non-FL (Figure 1B). Using flow cytometry and gating on “debris” population with very small forward scatter values, we verified that the extensive washing performed was sufficient to remove extracellular bacteria, as we detected sizeable mCherry^+^ signal in the “debris” population in samples without wash, but not in the samples prepared with washing (Figure S2). We also analyzed the infected macrophages using imaging flow cytometry (IFC) (Figure 2A). The results of this alternative analysis method were in very good agreement with those obtained from conventional flow cytometry, with more than 80% of the infected macrophages being mCherry^+^ (Figure 2B,C).

IFC allows distinguishing between intracellular and extracellular signal, unlike flow cytometry which only provides information on total fluorescence signal associated with a cell. We therefore used the internalization feature to quantify intracellular mCherry signal within the mCherry^+^ population in the IFC data. More than 95% of mCherry^+^ cells showed intracellular mCherry signal, as defined by an internalization score above 1, indicating that mCherry signal reports intracellular bacteria (Figure 2D). As flow cytometry and imaging flow cytometry data show comparable percentages of mCherry^+^ macrophages in the infected samples, this result indicates that both these methods allow sensitive detection of intracellular *M. abscessus*-mCherry bacteria. We then sought to validate this intracellular *M. abscessus* infection model for drug screens by testing the effect of clarithromycin and amikacin, antibiotics with known anti-*M. abscessus* activity [14], on the intracellular fluorescent bacteria.

### 2.2. Clarithromycin and Amikacin Treatment Reduce Intracellular M. abscessus Bacterial Load

Following the 4 h infection with *M. abscessus*-mCherry, the macrophages were incubated for 24 h with two concentrations of clarithromycin or amikacin, followed by analysis by flow cytometry and IFC. Treatment with 10 or 2.5 μM clarithromycin reduced the percentage of mCherry^+^ cells by approximately half, and significantly decreased the mCherry mean fluorescent intensity within the mCherry^+^ population (Figure 1B,C), as determined using both conventional flow cytometry and IFC. These results indicate that clarithromycin treatment in this infection model reduces both the proportion of infected cells, as well as the bacterial load within the infected macrophages.

Treatment with 10 μM amikacin, but not 2.5 μM amikacin, reduced the percentage of infected cells; and treatment with both 10 and 2.5 μM amikacin decreased the mCherry mean fluorescent intensity within the mCherry^+^ population (Figure 1B,C and Figure 2C), indicating that amikacin treatment reduces the proportion of infected cells and the bacterial load within the infected macrophages. Critically, both clarithromycin and amikacin treatment did not alter macrophage viability (Figure S3). We validated the flow cytometry and IFC data using colony forming units (cfu) plating, and we observed a reduction in bacterial load after antibiotics treatment (Figure S4).

We used equimolar concentrations of clarithromycin and amikacin in our experiments and found that clarithromycin was more potent in reducing the bacterial burden of macrophages. This result is consistent with the lower minimum inhibitory concentration of clarithromycin in broth culture of *M. abscessus* Bamboo (200 μM vs. 2 μM) [31]. Furthermore, clarithromycin (in contrast to amikacin) is known to accumulate in macrophages [32], which likely also contributes to the observed differential potencies of the two drugs. Together, our results show that the fluorescent *M. abscessus* infection model presented here provides a useful platform for anti-*M. abscessus* drug testing, simultaneously providing information on proportion of the infected cells, the intracellular load within the infected cells, as well as the viability of the infected cells.

### 2.3. Heterogeneity of Intracellular M. abscessus Infection

**Figure 3:**
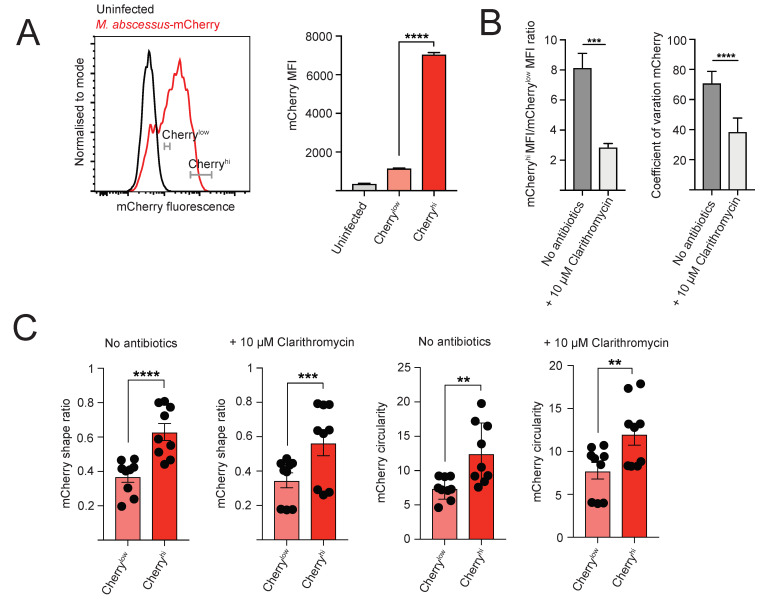
High variation in bacterial load in infected macrophages at single cell level. (**A**) Gating strategy used to define the 10% cells with lowest (mCherry^low^) and highest (mCherry^hi^) fluorescence within the mCherry^+^ population from conventional flow cytometry data. mCherry MFI on mCherry^low^ and mCherry^hi^ cells. Data from 1 experiment with three technical repeats, representative of three independent experiments. (**B**) Clarithromycin treatment reduces infection heterogeneity. Fold difference between mCherry MFI between mCherry^hi^ and mCherry^low^ populations from untreated and clarithromycin-treated cells. mCherry CV on mCherry^+^ populations from untreated and clarithromycin treated cells. Data from 2 independent experiments, with 3 technical repeats per experiment. (**C**) Effect of bacterial load on shape of subcellular regions containing intracellular bacteria. Shape ratio, circularity and compactness parameter values in mCherry^low^ and mCherry^hi^ populations from untreated or antibiotics treated macrophages analyzed by imaging flow cytometry. Data from 3 independent experiments, with 3 technical repeats per experiment. Unpaired *t*-test was used for analysis. ** = *p* ≤ 0.01, *** = *p* ≤ 0.001, **** = *p* ≤ 0.0001.

Unlike conventional cfu measurements, flow cytometry and IFC provide information about bacterial load at the single cell level for the infected macrophages. We therefore examined the heterogeneity of infection within the population of infected cells. To estimate the variation in bacterial load within the infected macrophage populations analyzed by conventional flow cytometry, we compared bacterial load (defined as the mCherry intensity) between cells with the lowest and highest 10% of mCherry signal within the mCherry^+^ population. The brightest 10% of population (Cherry^high^) had approximately 7-fold higher mCherry signal than the dimmest 10% population (Cherry^low^), thus demonstrating striking differences in intracellular bacterial load at the single cell level (Figure 3A). We observed similarly high levels of infection heterogeneity when using the human macrophage cell line THP-1 (Figure S5), indicating that our results are applicable to different host cell types.

Clarithromycin treatment decreased the infection heterogeneity at the single cell level. This was manifested by reduction in fold difference in mCherry mean fluorescent intensity between the Cherry^hi^ and Cherry^low^ populations after clarithromycin treatment (Figure 3B). Fluorescence signal heterogeneity can be quantified using coefficient of variation (CV), which reports the ratio of the width of fluorescent signal distribution and the mean fluorescence [33], and thus can be used as a measure of infection heterogeneity. Clarithromycin treatment decreased CV of mCherry fluorescence signal within the mCherry^+^ population, indicating reduced heterogeneity of infection (Figure 3B).

Moreover, IFC analysis revealed that cells from Cherry^hi^ and Cherry^low^ subpopulations showed differences in morphology of the subcellular regions containing the intracellular bacteria, with the Cherry^hi^ subpopulation displaying a more rounded phenotype, defined by IFC features such as shape ratio and circularity (Figure 3C). These differences between Cherry^hi^ and Cherry^low^ subpopulations were also observed for infected cells treated with clarithromycin.

**Figure 4:**
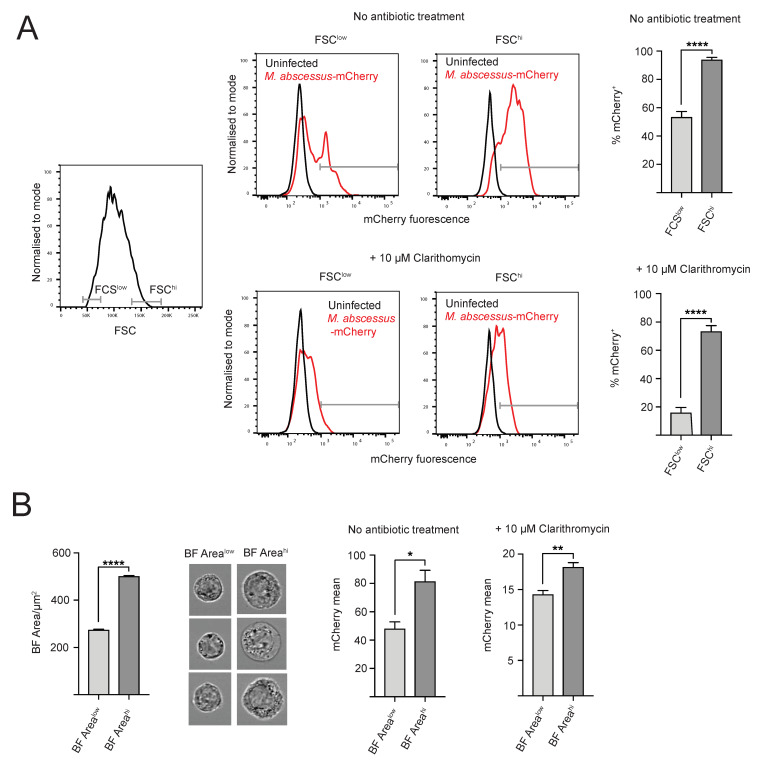
Host cell size correlates with bacterial load. (**A**) Conventional flow cytometry analysis of the influence of host cell size on % of infected host cells. Left: Gating strategy used to define cells with lowest (FCS^low^) and highest (FCS^hi^) size, defined based on FSC parameter. Right: Representative flow cytometry plots showing mCherry^+^ gate in FCS^low^ and FSC^hi^ subpopulations in untreated and clarithromycin-treated macrophages. Graph showing the % Cherry^+^ FSC^low^ and FSC^hi^ cells from untreated and clarithromycin-treated samples. Data from 3 independent experiments, with 3 experimental repeats per experiment. (**B**) Imaging flow cytometry analysis of the influence of host cell size on intracellular bacterial load within infected macrophages. Left: Brightfield (BF) area values for cells with 10–15% lowest (BF Area^low^) and highest (BF Area^hi^) values from population of mCherry^+^ macrophages infected with *M. abscessus*-mCherry. Representative BF images of cells from BF Area^low^ and BF Area^hi^ subpopulations. Right: Mean pixel (mCherry) on BF Area^low^ and BF Area^hi^ subpopulations from mCherry^+^ infected macrophage population from untreated and clarithromycin-treated samples. Data from 2 independent experiments, with 3 technical repeats per experiment. Unpaired *t*-test was used for analysis. * = *p* ≤ 0.05, ** = *p* ≤ 0.01, **** = *p* ≤ 0.0001.

This high level of heterogeneity in intracellular bacterial load can result from stochastic infection processes, or from underlying heterogeneity within the macrophage population. To distinguish between these two possibilities, we analyzed flow cytometry and IFC data to test if there is a correlation between intracellular bacterial load and overall cell morphology, on the basis of brightfield image. Based on flow cytometry data, we observed a correlation between macrophage size, defined using forward scatter (FSC) and percentage of infected cells (Figure 1B and Figure 2C). The FSC^hi^ population (10–15% of the live cells with the highest FSC values) has approximately two-fold higher percentage of mCherry^+^ positive cells, as compared to the FSC^low^ population (10–15% of the live cells with the lowest FSC values) (Figure 4A). We also observed a positive correlation between the host cell size and percentage of infected cells when human macrophage cell line THP-1 was used as the host cell line (Figure S5), suggesting that the observed effect is independent of host cell type.

We then tested if clarithromycin treatment, which reduces the overall percentage of infected cells (Figure 4A), can change the correlation between host cell size and percentage of infected cells. Interestingly, the effect of host cell size is even stronger for clarithromycin-treated cells, with the FCS^hi^ population having approximately three-fold higher percentage of mCherry^+^ cells then the FCS^low^ population. Moreover, clarithromycin treatment resulted in a stronger decrease in percentage of infected cells in FCS^low^ cells, indicating that clarithromycin treatment has biggest effect on FCS^low^ population.

We then sought to test if host cell size has an effect on intracellular bacterial load at the single cell level. We decided not to use conventional flow cytometry for this analysis, as the mean fluorescence intensity values obtained using this method report total fluorescence signal per cell, which is expected to increase with cell size. Instead, we used mean pixel fluorescence values from IFC data, in which the reported values are not influenced by size of the analyzed area. We observed correlation between macrophage size, as defined using brightfield mask area (BF Area) and mean mCherry mean pixel values, with bigger macrophages having significantly higher mCherry fluorescence (Figure 4B). This effect of macrophage size on intracellular bacterial load was also observed for clarithromycin-treated cells (Figure 4B). In summary, our data show high heterogeneity of intracellular bacterial load within the population of infected macrophages, with bigger cells having higher intracellular bacterial load.

## 3. Discussion

Use of flow cytometry and imaging flow cytometry to investigate *M. abscessus* macrophage infection revealed a very high degree of heterogeneity in terms of bacterial burden within the population of infected host cells. This was observed for infection of murine J774 and human THP-1 macrophage cell lines. Our assay was designed to minimize infection heterogeneity by use of a host cell line, rather than more complex populations of primary cells, and by using the high 10:1 MOI to reduce stochastic effects on the probability of a host cell being infected. However, even under these conditions, we observed heterogeneous infection outcomes, with a proportion of host cells not being infected, and with large differences in intracellular bacterial load between individual cells within the infected population. Moreover, the morphology of the intracellular *M. abscessus*-mCherry signal was highly variable at the single cell level, with mCherry^hi^ infected host cells showing more rounded morphology of the subcellular mCherry positive regions. Single cell heterogeneity of infection outcomes in host cells has been previously reported for several bacterial [26,27,34] and viral [35,36] pathogens, and better understanding of this phenomenon is important for design of improved therapeutic strategies that, for example, allow selective targeting of specific subpopulation of infected host cells. Critically, antibiotic treatment decreased heterogeneity within the infected host cell population.

Variation in intracellular bacterial burden at the single cell level can result from stochasticity of the infection process and/or from heterogeneity within the host cell population. Using flow cytometry and IFC, we have shown that, at the population level, a higher proportion of bigger host cells is infected with *M. abscessus* as compared to smaller host cells. Moreover, at the single cell level, infected bigger cells have higher intracellular bacterial burden than infected smaller cells. Similar effects of host cell size were shown previously for infections with vesicular stomatitis virus [35] and foot-and-mouth disease virus, but not for infections with influenza A virus [36]. This correlation between host cell size and intracellular bacterial load could reflect the effect of cell cycle stage on cell size, or cell metabolic fitness on cell size. These possibilities can be further investigated using the experimental system presented here, together with use of reporters of host cell metabolism and cell cycle stage. Interestingly, our data show that clarithromycin treatment has a stronger effect on small, rather than on large, host cells, suggesting that heterogeneity within host cell populations has an effect on antibiotic susceptibility of intracellular bacteria. Better understanding of *M. abscessus* infection at the single cell level is therefore critical for design of better therapeutic strategies.

A limitation of this study is that only one *M. abscessus* strain was used, *M. abscessus* Bamboo smooth morphotype. Future experiments will determine the effects of varying the bacterial strain on single cell infection heterogeneity. As *M. abscessus* smooth and rough variants are present in morphologically distinct phagosomes [37], it will be of particular interest to determine whether the bacterial morphotype influences infection behavior. Another limitation is use of macrophage lines as host cells. Although we validated the main findings from the murine J774 cell line using human macrophage cell line THP-1, the experimental system described here can be used to further investigate *M. abscessus* infection behavior in primary murine and human macrophages. Moreover, the experimental system described here can be subject to transcriptomics analysis to unravel the host and bacterial factors that drive the heterogeneity of *M. abscessus* infection.

## 4. Materials and Methods

### 4.1. Cell Culture

*M. abscessus* Bamboo was cultured in Middlebrook 7H9 media (Becton Dickinson, Franklin Lakes, NJ, USA) supplemented with 10% albumin-dextrose-catalase enrichment broth (Becton Dickinson, Franklin Lakes, NJ, USA), 0.5% glycerol and 0.05% Tween 80 (Sigma-Aldrich, St. Louis, MO, USA); hence referred to as complete 7H9 media. *M. abscessus* Bamboo is a smooth colony morphotype clinical isolate from a patient with amyotrophic lateral sclerosis and bronchiectasis. The strain was provided by Wei Chang Huang (Taichung Veterans General Hospital, Taichung, Taiwan) and was previously whole-genome sequenced which showed that it belongs to *M. abscessus* subsp. *abscessus* and harbors an inactive, clarithromycin-sensitive ermC28 sequevar [30].

J774 cells were cultured in DMEM (Gibco, Waltham, MA, USA) supplemented with 10% FBS, 100 U mL^−1^ penicillin and 10 mg mL^−1^ streptomycin, 292 mg mL^−1^ L-glutamine, 1 mM sodium pyruvate, 25 mM HEPES and 1 × MEM non-essential amino acids. The cells were passaged every 2–3 days.

THP-1 cells were cultured in RPMI (Gibco, Waltham, MA, USA) supplemented with 10% FBS, 100 U mL^−1^ penicillin and 10 mg mL^−1^ streptomycin, 292 mg mL^−1^ L-glutamine, 1 mM sodium pyruvate, 25 mM HEPES and 1 × MEM non-essential amino acids. The cells were passaged every 2–3 days.

### 4.2. Generation of M. abscessus-mCherry

mCherry was cloned into pMV306hsp using standard molecular biology techniques. pMV306hsp was a gift from Brian Robertson & Siouxsie Wiles (Addgene plasmid # 26155; http://n2t.net/addgene:26155; RRID:Addgene_26155). Prior to electroporation, frozen aliquots of *M. abscessus* Bamboo strain were thawed and re-suspended in complete 7H9 media at OD 0.05, followed by overnight growth to log phase at 37 °C, with shaking at 120 rpm. The pre-cultures were diluted to OD 0.1 using complete 7H9 to obtain 40 mL culture volume, and incubated at 37 °C in a roller apparatus at 100 rpm for 4 h. 4 mL of 2 M glycine was added before incubating the culture overnight under the same conditions. The next day, the cells were collected by centrifugation (10 min, 1900 rcf, 22 °C), washed three times each with 40 mL of wash buffer (10% glycerol, 0.02% tween), followed by re-suspension in 2 mL wash buffer. 200 µL of the bacteria was used for electroporation. Electroporation was performed with Gene Pulser Xcell™ Electroporation System (Bio-Rad, Hercules, CA, USA), and using a single pulse of 2.5 kV, 25 μF with the pulse-controller resistance set at 1000 Ω resistance. After electroporation, bacteria were immediately re-suspended in 10 mL complete 7H9 and cultured at 37 °C, followed by centrifugation and plating on 7H10 agar (Becton Dickinson, Franklin Lakes, NJ, USA) supplemented with 10% OADC Enrichment broth, 0.5% glycerol and 50 µg/mL kanamycin. The plates were grown for 1–2 weeks. Several colonies were selected for further testing, with measurement of OD and mCherry fluorescence performed using Infinite^®^ TECAN microplate reader. An isolate with good correlation between mCherry fluorescence and optical density was selected.

### 4.3. M. abscessus Infection of J774 and THP-1 Cells

24 h prior to infection, the host cells were passaged at 1:1 ratio. J774 cells were infected with *M. abscessus* at MOI 10:1 at 37 °C, 5% CO_2_ for 4 h, followed by three washes with PBS and subsequent culture at 37 °C, 5% CO_2_ for 24 h with or without the indicated antibiotics. THP-1 cells were infected with *M. abscessus* at MOI 10:1 at 37 °C, 5% CO_2_ for 24 h. For cfu plating, J774 cells were washed twice in PBS and re-suspended in 0.1% Triton X-100 in PBS. The cell lysates at 10^−3^, 10^−4^ and 10^−5^ dilutions were plated on 7H10 agar plates, with 50 μg/ mL kanamycin, followed by 1–2 weeks incubation at 37 °C and 5% CO_2_ and manual colony count.

### 4.4. Flow Cytometry Analysis

Cells were labelled using Live/Dead Fixable Green Dead Cell Stain (Invitrogen, Waltham, MA, USA) for 30 min on ice, followed by PBS wash and fixation with 4% paraformaldehyde (Fisher Scientific, Waltham, MA, USA) for 30 min at room temperature. The samples were then washed with PBS and analyzed using BD LSRFortessa X-20 flow cytometer. FlowJo V9 and V10 was used for flow cytometry data analysis. The following gating strategy was used for data analysis: cell signals were separated from that of debris based on FSC-A vs. SSC-A signal, single cells were gated based on SSC-A vs. SSC-H signal, live and dead cell populations were gated on the basis of green fluorescence signal, and mCherry^+^ gate was used on live single cells. This gate was set based on uninfected cells. The gating strategy to analyse the extracellular debris is shown in Figure S2. FlowJo V9 and V10 was used for flow cytometry data analysis.

### 4.5. Imaging Flow Cytometry Analysis

Samples were prepared as described above for flow cytometry analysis, with an additional DAPI labelling step for 10 min at room temperature after fixation (30). Samples were analyzed using AMNIS ImageStream^®^X Mark II Imaging Flow Cytometer. AMNIS Ideas software was used for data analysis, and FlowJo was used to generate the plots. The following gating strategy was used for data analysis: single cells were gated based on Area (Brightfield) vs. Aspect Ratio (Brightfield), then cells in focus were gated based on Gradient RMS (Brighfield), live cells were gated based on Intensity (Green), and mCherry+ gate was set using Mean Fixel (mCherry), based on uninfected cells. Ideas software default mask was used for analysis of mCherry mean pixel and morphology features, and Ideas software internalization wizard was used to analyze internalization of mCherry signal.

### 4.6. Statistical Analysis

Data was analyzed using GraphPad Prism, using unpaired *t*-test or one-way ANOVA, as indicated in the figure legends. ns = *p* > 0.05, * = *p* ≤ 0.05, ** = *p* ≤ 0.01, *** = *p* ≤ 0.001, **** = *p* ≤ 0.0001.

## 5. Conclusions

We describe here an experimental system for rapid and robust screening of compounds for their antimicrobial activity against intracellular *M. abscessus*. Our assay allows simultaneous analysis of multiple parameters that are not available from conventional screens, such as the proportion of infected host cells, average bacterial load per host cell from the infected population, and host cell viability. Our assay provides several advantages for drug screening over conventional cfu counting assays: (1) both the experimental setup as well as data acquisition and analysis are relatively fast and easy to perform, with no time consuming steps like growth of bacteria on plates and colony counting; (2) the assay takes into account the effect of host cells on the drugs tested, such that compounds with poor permeability or those metabolized very rapidly can be excluded from further analysis; (3) the assay allows monitoring of drug effects on host cell viability, thus providing a screen for drug toxicity on mammalian cells. Moreover, the assay is easily scalable into high-throughput format with the use of 96-well plates and high-throughput sampler mode on flow cytometers and/or imaging flow cytometers. We verified the suitability of this method for use in drug screening using two antibiotics with known anti-*M. abscessus* activity: clarithromycin and amikacin. The antibiotic treatments reduced the proportion of infected cells, as well as bacterial load within the infected macrophages. Importantly, both antibiotics had no effect on macrophage viability. These proof of principle experiments illustrate utility of the assay for drug screening experiments.

## Supplementary Materials

The following are available online at https://www.mdpi.com/2079-6382/9/10/711/s1, Figure S1: Time course analysis of macrophage viability post-infection, Figure S2: Post-infection washes remove extracellular bacteria, Figure S3: Clarithromycin and amikacin treatment reduce *M. abscessus* bacterial load, Figure S4: Amikacin and clarithromycin treatment does not alter macrophage viability, Figure S5: THP-1 infection with *M. abscessus*-mCherry.
