# Supplementary material for: Single Cell Analysis of Drug Susceptibility of Mycobacterium abscessus during Macrophage Infection
Source: Antibiotics (Basel). 2020 Oct 17;9(10):711. doi: 10.3390/antibiotics9100711 (PMC7650608; doi:10.3390/antibiotics9100711)
Supplement: Supplementary file 1 [file antibiotics-09-00711-s001.zip › supplementary/Supplementary Figure Legends.pdf]

Supplementary Figure Legends

Supplementary Figure 1. Time course analysis of macrophage viability post-infection. J774 macrophages were infected with FL or non-FL *M. abscessus*, followed by washing and incubation for 24, 48 or 72h. Cell viability was assessed using flow cytometry with live/dead staining. Data from 1 experiment, with 3 technical replicates per samples.

Supplementary Figure 2. **Post-infection washes remove extracellular bacteria.** J774 cells were infected with M. abscessus-mCherry or non-FL *M. abscessus*. The + wash samples were washed 3x with PBS after 4h, whereas the –wash samples were not subject to washing. 24h later, the cells were analysed by flow cytometry to detect extracellular mCherry+ bacteria within the "debris" gate. The FSC vs SSC plot shows the gating strategy used to define the "debris" and "macrophage" gates. Representative flow cytometry plots showing mCherry fluorescence within the "debris" gate after infection with *M. abscessus*-mCherry or non-FL *M. abscessus*. The graph shows % of mCherry+ events in the "debris" gate from the indicated samples. Data from 1 experiment with 2 technical repeats (-wash samples), or 3 experiments with 3 technical repeats (+ wash samples).

Supplementary Figure 3. **Clarithromycin and amikacin treatment reduce *M. abscessus* bacterial load.** J774 macrophages were infected with M. abscessus-mCherry for 4 hours, followed by 24h culture with or without the indicated antibiotics for 24h, cell lysis and plating for CFU counts.

Supplementary Figure 4. Amikacin and clarithromycin treatment does not alter macrophage viability. J774 macrophages were infected with *M. abscessus*-mCherry for 4h, followed by 24h culture with or without the indicated antibiotics. The % live macrophages was then determined using live/dead staining and flow cytometry. Data from 3 independent experiments with 3 (*M. abscessus*-mCherry infection, *M. abscessus*-mCherry infection + clarithromycin) or 2 (uninfected) technical replicates, or from 1 experiment with 3 technical replicates (*M. abscessus*-mCherry infection + amikacin).

Supplementary Figure 5. THP-1 infection with *M. abscessus* - mCherry. Representative flow cytometry plots showing mCherry fluorescence of THP-1 human macrophage cell line infected with M. abscessus - mCherry or non-FL *M. abscessus*. mCherry MFI on mCherry^{low} (bottom 10-15% mCherry fluorescence) and mCherry^{hi} populations (top 10-15% mCherry fluorescence). Data from 1 experiment with 4 technical repeats, representative of 2 independent experiments. Graph showing the % Cherry+ cells from FSC^{low} (bottom 10-15% FSC values) and FSC^{hi} (top 10-15% FSC values) populations. Data from 2 independent experiments, with 3 technical repeats.
