# Supplementary figures and images for: Single Cell Analysis of Drug Susceptibility of Mycobacterium abscessus during Macrophage Infection
Source: Antibiotics (Basel). 2020 Oct 17;9(10):711. doi: 10.3390/antibiotics9100711 (PMC7650608; doi:10.3390/antibiotics9100711)

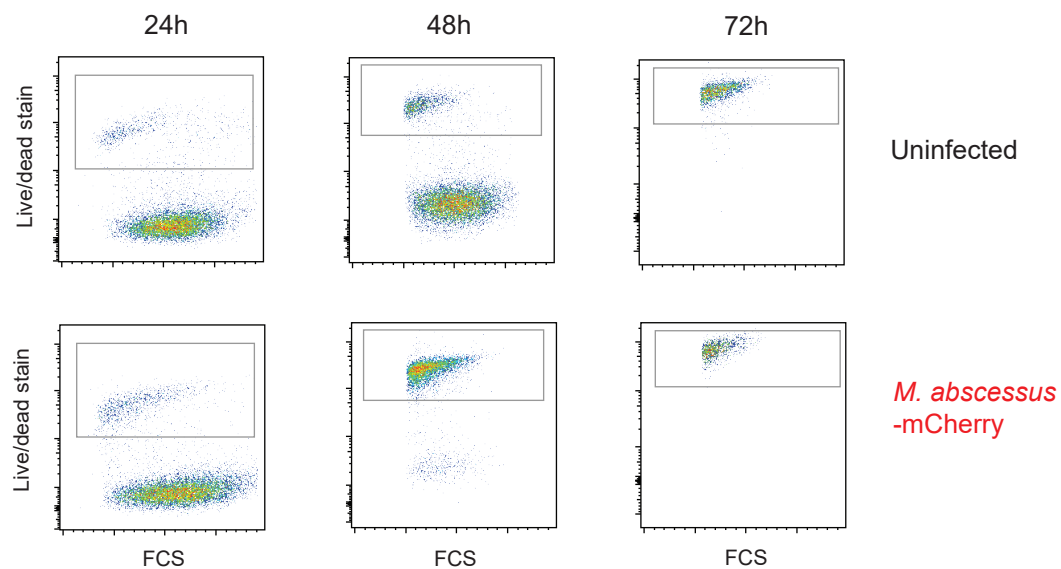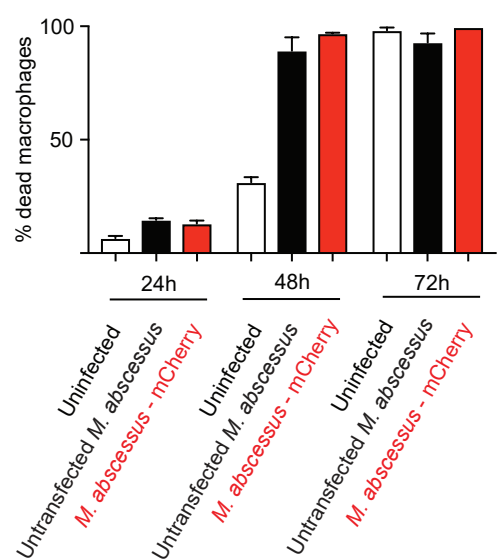

Supplement: Supplementary file 1 [file antibiotics-09-00711-s001.zip › supplementary/Supplementary Figure 1.pdf]

Untransfected *M. abscessus*  
*M. abscessus* - mCherry

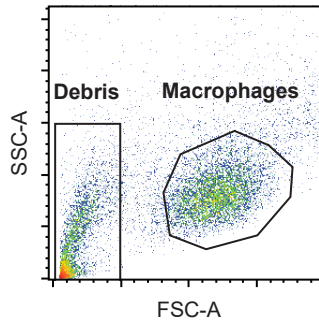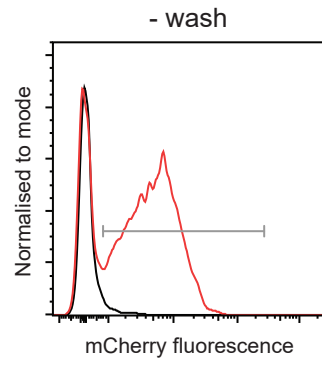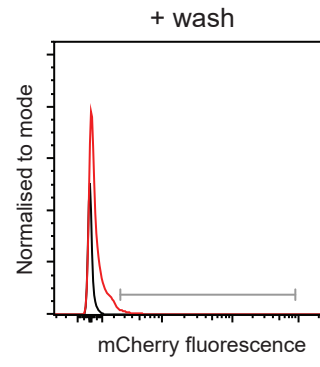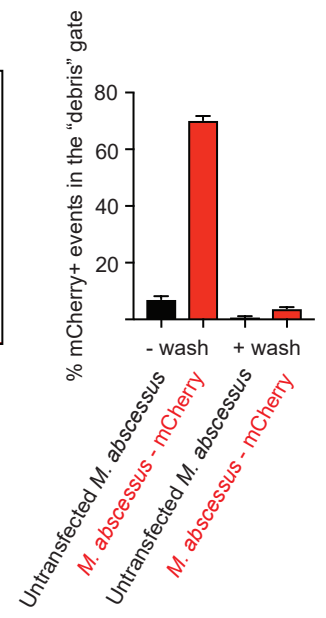

Supplement: Supplementary file 1 [file antibiotics-09-00711-s001.zip › supplementary/Supplementary Figure 2.pdf]

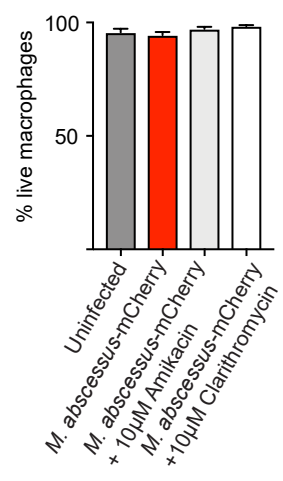

Supplement: Supplementary file 1 [file antibiotics-09-00711-s001.zip › supplementary/Supplementary Figure 3.pdf]

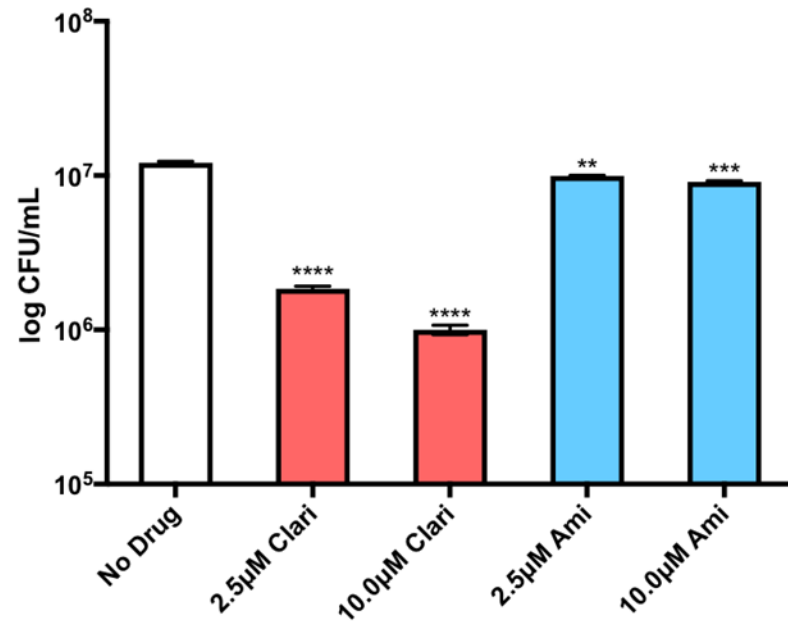

Supplement: Supplementary file 1 [file antibiotics-09-00711-s001.zip › supplementary/Supplementary Figure 4.pdf]

Untransfected *M. abscessus*  
*M. abscessus* - mCherry

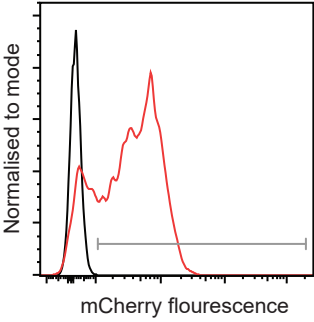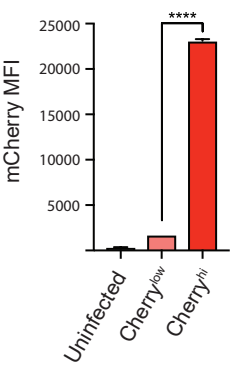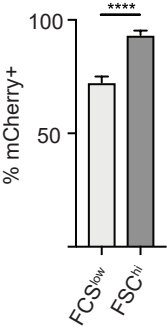

Supplement: Supplementary file 1 [file antibiotics-09-00711-s001.zip › supplementary/Supplementary Figure 5.pdf]
